# Commentary on Urinary l-erythro-β-hydroxyasparagine: a novel serine racemase inhibitor and substrate of the Zn^2+^-dependent d-serine dehydratase

**DOI:** 10.1042/BSR20211524C

**Published:** 2021-12-07

**Authors:** Fabio K. Tamaki

**Affiliations:** Drug Discovery Unit, School of Life Sciences, University of Dundee, Dow Street, Dundee DD1 5, U.K.

**Keywords:** d-serine, amino acids, serine racemase

## Abstract

The analysis of the urine contents can be informative of physiological homoeostasis, and it has been speculated that the levels of urinary d-serine (d-ser) could inform about neurological and renal disorders. By analysing the levels of urinary d-ser using a d-ser dehydratase (DSD) enzyme, Ito et al. (*Biosci. Rep.*(2021) **41**, BSR20210260) have described abundant levels of l-erythro-β-hydroxyasparagine (l-β-EHAsn), a non-proteogenic amino acid which is also a newly described substrate for DSD. The data presented support the endogenous production l-β-EHAsn, with its concentration significantly correlating with the concentration of creatinine in urine. Taken together, these results could raise speculations that l-β-EHAsn might have unexplored important biological roles. It has been demonstrated that l-β-EHAsn also inhibits serine racemase with *K*_i_ values (40 μM) similar to its concentration in urine (50 μM). Given that serine racemase is the enzyme involved in the synthesis of d-ser, and l-β-EHAsn is also a substrate for DSD, further investigations could verify if this amino acid would be involved in the metabolic regulation of pathways involving d-ser.

## Commentary

The alphabet of standard 20 l-amino acids constitutes the biological building block for the construction of proteins in all organisms. The reasons for the almost exclusive use of l-amino acids to build proteins is not fully understood yet [1]. Besides the biological importance of l-amino acids, an extensive number of other non-proteinogenic amino acids, including d-amino acids [2,3], have been associated with diverse biological roles in the past years. One example of physiologically important d-amino acid is d-serine (d-ser), a neuromodulator constituting ∼25% of the total serine in the central nervous system and involved in the glutamatergic neurotransmission [4].

As a consequence of the strong evolutionary bias of living systems towards the use of l-amino acids for protein synthesis, only specific enzymes can recognise d-amino acids, as exemplified by d-amino acid transaminase (DAAT), which converts d-Alanine into other d-amino acids; serine racemase, responsible for the conversion of l-ser into d-ser; and d-ser dehydratase (DSD) and d-amino acid oxidase, both involved in the degradation of d-Ser.

In previous works, the authors used the enzyme DSD from *Saccharomyces cerevisiae* (Dsd1p) to analyse the levels of d-ser and l-ser as biomarkers in urine and plasma. These works also resulted in the finding of a new unknown substrate for Dsd1p in urine. In this paper [5], Ito *et al*. initially found that the new Dsd1p substrate is the amino acid β-hydroxyl asparagine. For this, they have analysed the amino acid contents in urine using a cascade of coupled reactions employing enzymes that recognise d-amino acids (Dsd1p and DAAT). Mass spec analysis of the amino acid derivatised with 6-aminoquinolyl-carbamyl further supported it is β-hydroxyl asparagine. To resolve the chiral form of β-hydroxyl asparagine, this amino acid was converted into β-hydroxy aspartate for retention time comparisons to commercial stereoisomers, which revealed the chiral form of the urinary amino acid as l-erythro-β-hydroxyasparagine (l-β-EHAsn).

Dsd1p has preference for d-amino acids as substrate, and the binding mechanism of its newly described substrate l-β-EHAsn into the enzyme active site has been speculated. Dsd1p shares 25% identity with d-threo-3-hydroxyaspartate dehydratase (d-THA-DH) from *Delftia* sp., a stereospecific enzyme able to use both amino acids d-threo-β-hydroxyaspartate and l-β-EHAsp as substrates. Given the identity between enzymes and their capacity of using the respective l-β-EH ASX as substrate, an *in-silico* 3d-structural model of Dsd1p was built using the crystal structure of d-THA-DH complexed with l-β-EHAsp as template. The generated 3D model suggests a correct coordination for binding of l-β-EHAsn in the active site of Dsd1p, leading to further speculations of a possible mechanism of α, β-elimination of l-β-EHAsn. Worth emphasising that, although automated *in-silico* modelling tools have greatly improved in past years, 3D models should be carefully used to determine the binding of compounds, as they are likely to show introduction of folding bias from the template (d-THA-DH complexed with l-β-EHAsp). Thus, a good positioning of l-β-EHAsn in the active site of the Dsd1p 3D model could reflect the bias construction of the model instead of giving realistic insights into the enzyme specificity for l-β-EHAsn. The use of molecular dynamics or docking would be more appropriate for generating binding information of l-β-EHAsn into the active site of Dsd1p (though less trustworthy in a 3D model).

The presence of l-β-EHAsn in the urine was described over 50 years ago. However, little attention has been paid to this amino acid, despite its presence at high concentrations in urine (∼50 μM, similar to other proteinogenic amino acids). It was demonstrated that l-β-EHAsn is present only in the urine of rats, but not in the plasma, cerebrum, cerebellum, kidneys, liver or testis; and only present at negligible levels in the diet of the rats, suggesting its endogenous production. Combined with a tight correlation between the levels of l-β-EHAsn (but not l-Asn) and creatinine in the urine, these results allow to speculate that l-β-EHAsn might have important biological significance. However, possible biological roles of l-β-EHAsn have been underexplored here and is certainly an interesting field for further investigation.

Lastly, based on the structural similarity between l-β-EHAsn and l-β-EHAsp, it has been speculated that the former could also inhibit serine racemase, an enzyme involved in the production of endogenous d-ser and inhibited by the latter [6]. Mode of inhibition studies for serine racemase have shown that l-β-EHAsn is a competitive inhibitor for l-Ser, with inhibition constant values (*K*_i_: 40 μM) similar to its concentration found in the urine (50 μM). Taken together the degradation of l-β-EHAsn by Dsd1p and its inhibitory effects on serine racemase, results suggest the involvement of l-β-EHAsn in metabolic regulation of pathways related d-ser. Unfortunately, it has not been shown that l-β-EHAsn inhibits human serine racemase, a more relevant model; if this inhibition is proven true, modified versions of l-β-EHAsn could serve as new start points for drug discovery programs targeting human serine racemase [7].

The efforts of Ito *et al*. expand the knowledge of non-proteinogenic amino acids, with the characterisation of the endogenous urinary l-β-EHAsn as a substrate for Dsd1p. The results presented open new avenues to understanding the biological importance of l-β-EHAsn and its possible involvement in signalling, metabolism, and neurotransmission. The description of a chemical route for the synthesis of l-β-EHAsn will likely help its production as a chemical tool to further understand its biological roles and support future medicinal chemistry efforts to find new inhibitors of serine racemases.

## Abbreviations

DAAT: d-amino acid transaminase
DSD: d-serine dehydratase
d-ser: d-serine
d-THA-DH: d-threo-3-hydroxyaspartate dehydratase
l-beta-EHAsn: l-erythro-β-hydroxyasparagine
